# Threatened Miscarriage

**Published:** 1893-05

**Authors:** W. S. Lane

**Affiliations:** House Surgeon, Homœopathic Hospital, Toronto


### THREATENED MISCARRIAGE.

W. S. LANE, M. D., HOUSE SURGEON, HOMŒOPATHIC HOSPITAL, TORONTO.

Knowing that the editor of THE HOMŒOPATHIC PHYSICIAN is always looking for information for that valuable journal I thought that perhaps the following case might be found of some value.

I might say, that being an allopath when I took the position here as house surgeon last May, I have not yet become readily conversant with the homœopathic materia medica, so this case seemed to me of more that usual interest inasmuch as I could view it from the standpoint of almost a veritable sceptic. It also convinced me of the efficacy of the *high potency* as a material and quick-acting remedy and its superiority to the low where rapid and deep measures are required.

Mrs. A——, æt. twenty-three, a blonde, Danish extraction, primipara, came to the hospital October 30th, at ten o'clock in the evening. A glance at her told me of the intense pain she was suffering. Upon questioning her I could elicit nothing, as she could talk no English and I no Danish, but fortunately her husband was with her, and, in broken English, he gave me the following: She was six months pregnant. The day before he had given her two cathartic pills for the purpose of relieving her very constipated bowels, and that day (October 30th) she had a slight movement which caused her some pain. At the same time she felt a discharge from vagina, and, on examination, found it of a sanguinous character, causing the parts to have a burning hot feeling. This did not alarm her until that evening when it got more copious, and pains—slight, however—came in the region of the uterus. They came and went, but not being at all severe caused her no uneasiness. At nine o'clock they came on in earnest, and her back, in a short time, felt as if it were breaking in two.

I had her taken to a private ward in the maternity department and put to bed, and on examination found a considerable bloody, hot discharge, the uterus quite down in the pelvis, and the os uteri dilated enough to enter the point of my index finger. She complained also of very severe bearing-down pains starting in the back—low down—running round forward and pressing toward the vulva, causing a sensation as if all the internal organs were going to be pressed through. Her face was very flushed, eyes quite red or inflamed, carotids throbbing, pulse rapid, full, hard, and tense, pupils dilated, and the head in general very hot. With this information, I went to the telephone and sent a message to the physician of the week, who unfortunately could not attend, and asked his advice as to the remedy. He prescribed *Belladonna*$^{12x}$, repeated every fifteen minutes until action took place, then to stop.

She was given the first dose at eleven o'clock, and repeated every quarter of an hour until twelve, but instead of benefit the pain kept gradually increasing in quality and quantity; the os was now dilated so as to admit my two fingers tightly squeezed together, and with every pain the presenting part was felt to press firmly upon the point of my finger, and then as the pain relaxed to recede. Thinking that perhaps my inexperience in Homœopathy would not allow me to note with that acumen necessary in such cases any fine action of the drug, whether amelioration or aggravation, I was afraid lest I had drugged too much, so only repeated half-hourly until one o'clock, but to no avail. At one another examination was made, as the patient seemed to have more continued and stronger pains. The os was found dilated enough to readily admit my three fingers, and with every pain the membranes were felt to bulge through. All this time the discharge was more bloody and profuse.

I, of course, at this stage, thought the case hopeless, but as the low potency was not evidently doing the work cut out for it I concluded as a last resort to run chances of incurring the anger of the physician and give on my own authority a high potency. Taking from my small pocket-case a most reliable potency of the drug, 40m, I gave a single dose on the tongue, and then went on with my preparations for emergency and premature labor. Fifteen minutes passed, and apparently no change; things seemed about at a standstill, but at the half-hour, what a difference! Pains were weak and far apart, os thoroughly contracted, no discharge, and patient quiet and sleepy. I left the room, and asked the nurse to carefully watch her, and in ten minutes returned to find patient sleeping soundly and quietly, which she did all night, awaking at eight o'clock next morning with no pain, feeling fresh and bright and quite comfortable.

She remained in the hospital six days, during which time there was not a return of a single symptom—no pain; os quite firm and contracted, and the bowels moving naturally.

This case I do not report as a mere case of threatened miscarriage, but to show what seemed to me the power and value of a high potency drug in a case requiring *somewhat* RAPID action. The low potency was given by myself in single-drop doses on the tongue, so had the best chance for quick action. I have not the slightest doubt that had I given the high drug in the first place I should have got to bed at twelve instead of two. No doubt also that it would have acted much more rapidly had it not been partially antidoted by the low potency in the beginning.

